# Bacteroides thetaiotaomicron Outer Membrane Vesicles Modulate Virulence of Shigella flexneri

**DOI:** 10.1128/mbio.02360-22

**Published:** 2022-09-14

**Authors:** Nicholas L. Xerri, Shelley M. Payne

**Affiliations:** a Department of Molecular Biosciences, The University of Texas at Austin, Austin, Texas, USA; University of Hawaii at Manoa

**Keywords:** *Shigella flexneri*, *Bacteroides thetaiotaomicron*, microbiota, outer membrane vesicles, *Shigella*, enteric pathogens, gut microbiome, intracellular pathogen, virulence regulation

## Abstract

The role of the gut microbiota in the pathogenesis of Shigella flexneri remains largely unknown. To understand the impact of the gut microbiota on S. flexneri virulence, we examined the effect of interspecies interactions with Bacteroides thetaiotaomicron, a prominent member of the gut microbiota, on S. flexneri invasion. When grown in B. thetaiotaomicron-conditioned medium, S. flexneri showed reduced invasion of human epithelial cells. This decrease in invasiveness of S. flexneri resulted from a reduction in the level of the S. flexneri master virulence regulator VirF. Reduction of VirF corresponded with a decrease in expression of a secondary virulence regulator, *virB*, as well as expression of S. flexneri virulence genes required for invasion, intracellular motility, and spread. Repression of S. flexneri virulence factors by B. thetaiotaomicron-conditioned medium was not caused by either a secreted metabolite or secreted protein but rather was due to the presence of B. thetaiotaomicron outer membrane vesicles (OMVs) in the conditioned medium. The addition of purified B. thetaiotaomicron OMVs to S. flexneri growth medium recapitulated the inhibitory effects of B. thetaiotaomicron-conditioned medium on invasion, virulence gene expression, and virulence protein levels. Total lipids extracted from either B. thetaiotaomicron cells or B. thetaiotaomicron OMVs also recapitulated the effects of B. thetaiotaomicron-conditioned medium on expression of the S. flexneri virulence factor IpaC, indicating that B. thetaiotaomicron OMV lipids, rather than a cargo contained in the vesicles, are the active factor responsible for the inhibition of S. flexneri virulence.

## INTRODUCTION

Shigella flexneri is an enteric pathogen that causes bacillary dysentery in humans (1). After being ingested, S. flexneri passes through the stomach and small intestine before entering the colon, where the expression of invasion genes (2) carried on its virulence plasmid (3) enables it to invade the colonic epithelium (4). Coordination of invasion, vacuole lysis, and intercellular spread by S. flexneri relies on the timed expression of a complex array of virulence genes (5). The genes involved in this virulence process are controlled by a master transcriptional regulator, VirF, which directly regulates *icsA*, a virulence gene encoding a protein necessary for S. flexneri motility within host cells, and *virB*, a gene encoding a secondary transcriptional regulator required for virulence (6, 7). VirB, in turn, regulates a variety of type three secretion system (T3SS) and invasion genes such as *ipaA*, *-B*, *-C*, and -*D* (8).

Prior to gaining access to the intestinal epithelium for invasion, S. flexneri encounters a variety of potential obstacles in the colon, including the trillions of established bacteria that make up the human gut microbiota (9). However, the role of the gut microbiota in S. flexneri pathogenesis remains largely unknown. While conventional guinea pigs and mice are resistant to colonization by S. flexneri, germfree animals are susceptible to colonization, indicating a possible role for the microbiota in resistance to infection (10). Monoassociating germfree animals with Escherichia coli prior to challenge with S. flexneri restores resistance to S. flexneri colonization, but monoassociating mice with *Bacteroides*, a prominent member of the human gut microbiota, has no effect on S. flexneri colonization. Interestingly, diassociating mice with both E. coli and *Bacteroides* has the largest impact on S. flexneri colonization, causing S. flexneri to decrease to undetectable levels in the colon (10–12). Together, these studies suggest that the gut microbiota may function in preventing S. flexneri infection of the colon; however, since E. coli is often only a minor constituent of the human gut microbiota (13), and small-animal models fail to recapitulate many aspects of shigellosis in humans (14), the relevance of these data for S. flexneri infection in humans is unclear.

Another bacterium found in the gut, *Lactobacillus*, has been of interest to researchers as a probiotic due to its inhibitory effects on enteric pathogens (15). Consistent with the possibility that *Lactobacillus* is protective against *Shigella*, a metagenomic study looking at the association between gut microbiota composition, *Shigella* levels, and diarrheal status in children in low-income countries found that children who were colonized by certain species of *Lactobacillus* had moderate-to-severe diarrhea less often than expected when they were also colonized by *Shigella* (16). This possible role for *Lactobacillus* spp. in preventing symptomatic S. flexneri infection is further supported by experiments using cell culture models that demonstrate that *Lactobacillus* spp. can inhibit *Shigella* attachment to and invasion of colonic epithelial cells (17–19). However, similarly to E. coli, *Lactobacillus* spp. are minor constituents of the gut microbiota (20). Furthermore, only a subset of species of *Lactobacillus* has been observed to stably colonize the intestinal tract, while others are known to only transiently colonize the gut (21). Our understanding of the interactions between S. flexneri and prominent members of the gut microbiota and what effects these have on S. flexneri virulence remains limited.

The gut microbiota is an incredibly diverse community comprised of hundreds of bacterial species. Most of these bacteria belong to just two phyla, *Bacteroidota* and *Bacillota* (formerly called *Bacteroidetes* and *Firmicutes*, respectively) (22). While a number of genera from *Bacillota* can be found in the human gut microbiota, *Bacteroidota* are largely represented by just four genera, the most abundant of which are *Bacteroides* and *Prevotella* (23). In humans, there appears to be a tradeoff between having a *Prevotella* dominated microbiota and having a *Bacteroides*-dominated one (24–26), with studies suggesting that diet may play a fundamental role in enriching for either *Prevotella*, which is more abundant in people of non-Western societies, or *Bacteroides*, which is more abundant in people of Western societies (27–30). In people from the United States and Western Europe, *Bacteroides* is often the most abundant genus in the gut microbiota (24, 31). Because they are major constituents of the human gut microbiota and are prevalent among humans from different populations, *Bacteroides* species have been proposed as model organisms for studying gut microbes (23).

One of the best-studied *Bacteroides* species, Bacteroides thetaiotaomicron, has been shown to impact the virulence of a number of enteric pathogens (32). B. thetaiotaomicron releases succinate that increases virulence gene expression (33), proteases that cleave the T3SS (34), and fucose that represses T3SS gene expression of enterohemorrhagic E. coli (35). Additionally, B. thetaiotaomicron polysaccharides have been shown to inhibit toxin release by Clostridium difficile (36). In this study, we examine the effects of B. thetaiotaomicron on S. flexneri virulence. We show that B. thetaiotaomicron suppresses S. flexneri invasion through the downregulation of S. flexneri T3SS gene expression. This suppression is mediated by B. thetaiotaomicron outer membrane vesicles (OMVs), which fuse to S. flexneri and posttranscriptionally repress its master virulence regulator, VirF. This demonstrates that a prominent member of the human gut microbiota impacts *Shigella* pathogenesis and that OMVs can modulate the gene expression of enteric pathogens.

## RESULTS

### Bacteroides thetaiotaomicron-conditioned medium inhibits Shigella flexneri invasion.

**FIG 1:**
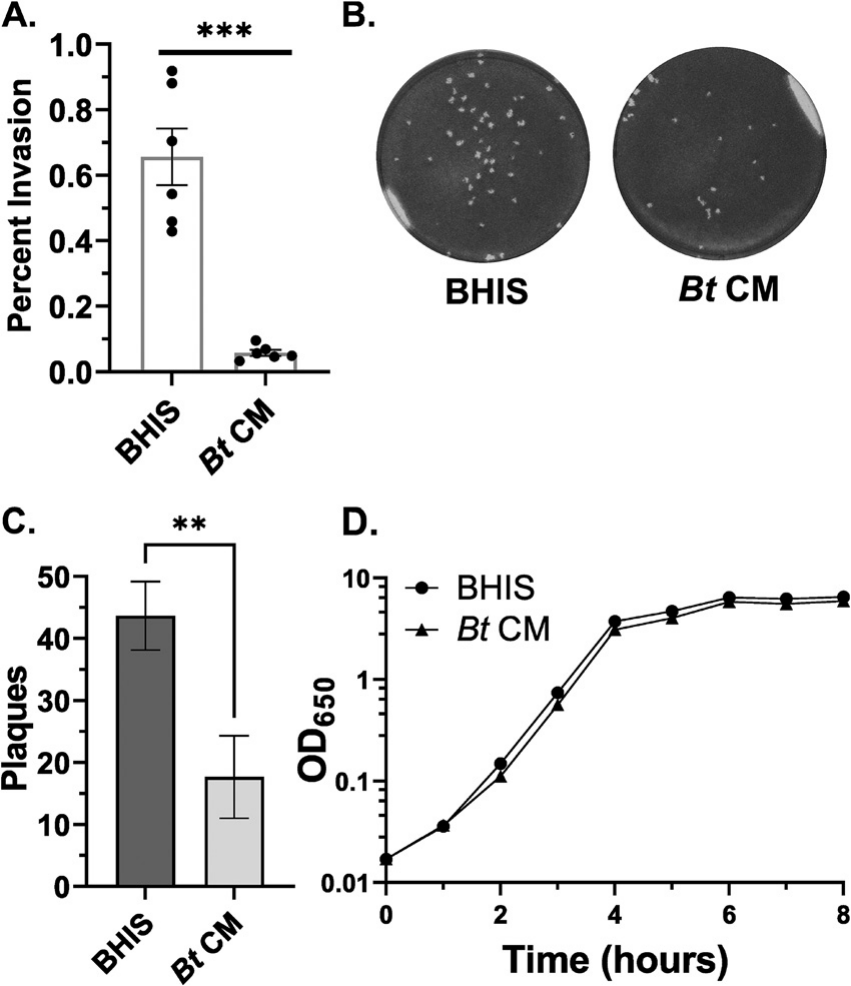
B. thetaiotaomicron CM impacts S. flexneri virulence, but not growth, during log phase. Samples labeled B. thetaiotaomicron (*Bt*) CM were grown in a mixture of one-half BHIS and one-half B. thetaiotaomicron CM. (A) Invasion rates of S. flexneri grown in BHIS or B. thetaiotaomicron CM as measured by a gentamicin protection assay are significantly different (0.66% versus 0.06%, respectively; *P* = 0.0008, *n* = 6). Error bars indicate the standard error of the mean calculated from six biological replicates. *P* values were determined by a paired, two-tailed *t* test. (B) A confluent layer of Henle cells was infected with S. flexneri grown in BHIS or B. thetaiotaomicron CM. For visualization of plaques, Henle cell monolayers were stained after 72 h of infection. (C) Average numbers of plaques formed by S. flexneri after growth in BHIS and after growth in B. thetaiotaomicron CM were significantly different (43.7 versus 17.7, respectively; *P* = 0.003) as measured from three biological replicates. Error bars indicate standard deviation. *P* values were determined using a paired, two-tailed *t* test. (D) Growth curves of S. flexneri in BHIS and B. thetaiotaomicron CM are derived from three biological replicates. *P* values of doubling times were determined using a paired, two-tailed *t* test.

To determine whether interspecies interactions with members of the human gut microbiota impact S. flexneri virulence, we studied the effect that the common gut microbe B. thetaiotaomicron has on S. flexneri invasion and cell-to-cell spread (plaque formation) in a monolayer of cultured epithelial cells. We simulated S. flexneri encountering an established gut microbiota by growing S. flexneri in cell-free conditioned medium (CM) collected from late-stationary-phase B. thetaiotaomicron cultures. S. flexneri grown in brain heart infusion (BHIS) supplemented with B. thetaiotaomicron CM had an 11-fold reduction in invasion rate relative to S. flexneri grown in BHIS alone (Fig. 1A). Additionally, S. flexneri grown in the presence of B. thetaiotaomicron CM formed fewer plaques than S. flexneri grown in BHIS alone (Fig. 1B and C), consistent with the lower rate of invasion. Together, this indicates that B. thetaiotaomicron releases a factor that inhibits S. flexneri invasion.

Because it is possible that the observed decrease in invasiveness was due to effects of B. thetaiotaomicron CM on S. flexneri growth rate, we measured the growth of S. flexneri in the presence and absence of B. thetaiotaomicron CM. The only effect noted on S. flexneri growth rate by B. thetaiotaomicron CM was during the first 2 h of growth, when the doubling time of S. flexneri grown in BHIS supplemented with B. thetaiotaomicron CM (37.6 min) was significantly longer than its doubling time in BHIS alone (29.2 min, *P* = 0.022, Fig. 1D). However, during mid- and late log phase, the growth phase used for the invasion assay, the doubling times of S. flexneri in the presence and absence of B. thetaiotaomicron CM were nearly identical (25.7 min versus 25.9 min, *P* = 0.64, Fig. 1D), indicating that the decrease in S. flexneri invasion following growth in B. thetaiotaomicron CM was not due to an effect on growth.

### B. thetaiotaomicron CM reduces the levels of S. flexneri virulence factors.

**FIG 2:**
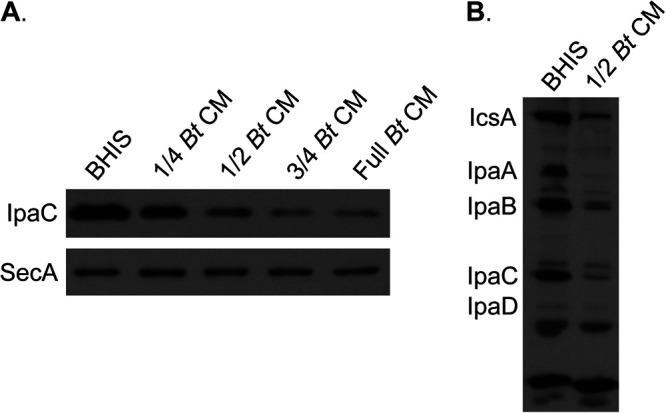
B. thetaiotaomicron CM reduces S. flexneri virulence protein levels. (A) Western blotting using anti-IpaC antisera was performed on total proteins collected from S. flexneri grown to log phase in BHIS supplemented with increasing proportions of B. thetaiotaomicron CM. Anti-SecA antiserum was used as a loading control. (B) Western blotting using monkey anti-S. flexneri convalescent-phase antiserum was performed to look at the protein levels of a panel of S. flexneri virulence factors when grown in either BHIS or a mixture of one-half BHIS and one-half B. thetaiotaomicron CM. For both panels, representative Western blots from 3 independent experiments are shown.

Invasion of eukaryotic cells by S. flexneri is type three secretion system (T3SS) dependent. Since growth of S. flexneri in the presence of B. thetaiotaomicron CM resulted in reduced invasiveness of S. flexneri (Fig. 1A), we hypothesized that B. thetaiotaomicron CM represses S. flexneri T3SS protein expression. To test this, S. flexneri was grown in BHIS supplemented with increasing proportions of B. thetaiotaomicron CM, and the amount of the S. flexneri T3SS protein IpaC was determined by Western blotting. B. thetaiotaomicron CM inhibited the expression of IpaC in a dose-dependent manner (Fig. 2A). This repression was not unique to IpaC. Other T3SS proteins (IpaA, IpaB, and IpaD) and a non-T3SS-associated virulence factor (IcsA) were also produced in smaller amounts when S. flexneri was grown in the presence of B. thetaiotaomicron-conditioned medium (Fig. 2B). In addition to cell-associated virulence factors, secreted virulence factors, including IcsA, IpaA, IpaB, and IpaC, were also decreased in the presence of B. thetaiotaomicron CM relative to BHIS (see Fig. S1 in the supplemental material), suggesting that B. thetaiotaomicron CM reduces the production of S. flexneri virulence factors rather than triggering their secretion.

10.1128/mbio.02360-22.1 FIG S1 B. thetaiotaomicron CM reduces S. flexneri virulence factor secretion. Secreted proteins were collected from S. flexneri grown in either BHIS or one-half B. thetaiotaomicron CM. A Western blot assay using monkey anti*-Shigella* convalescent-phase antiserum was performed to look at the relative secretion of a panel of virulence factors. A representative Western blot from 5 independent replicates is shown. Download FIG S1, DOCX file, 0.5 MB. Copyright © 2022 Xerri and Payne. 2022 Xerri and Payne. https://creativecommons.org/licenses/by/4.0/ This content is distributed under the terms of the Creative Commons Attribution 4.0 International license.

### B. thetaiotaomicron CM reduces S. flexneri virulence genes at the transcriptional and posttranscriptional level.

**FIG 3:**
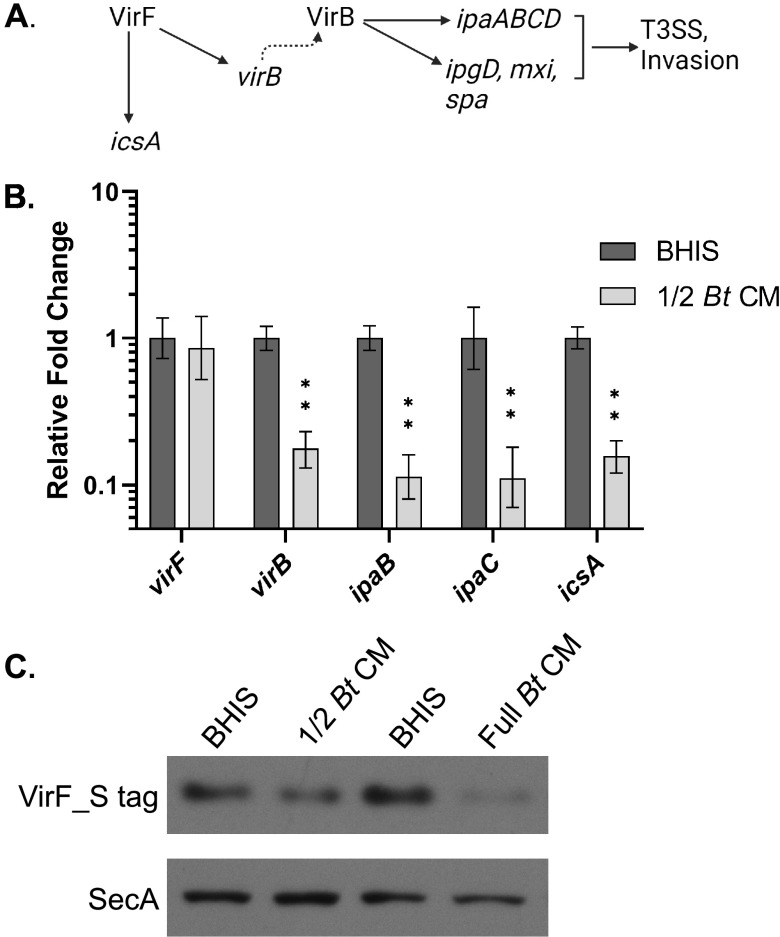
B. thetaiotaomicron CM transcriptionally represses S. flexneri virulence gene expression and posttranscriptionally represses the master virulence regulator VirF. (A) Schematic of the virulence gene regulon of S. flexneri. Illustration created with BioRender.com. (B) Relative virulence gene expression of S. flexneri grown to log phase in either BHIS or one-half B. thetaiotaomicron CM was measured by RT-qPCR. *C_T_* values were normalized to the mean for two endogenous controls, *gyrA* and *secA*. *P* values were determined from the Δ*C_T_* values of three biological replicates using a two-tailed Student *t* test with the Holm-Šidák correction for multiple comparisons. (**, *P* < 0.01). Error bars indicate standard deviation. (C) Total proteins were collected from S. flexneri expressing *virF_S-tag* from the native *virF* promoter on the plasmid pWKS30 grown under the indicated conditions. Western blotting was performed on these proteins using an anti-S peptide epitope tag antibody to look at relative VirF_S-tag protein levels. Anti-SecA antiserum was used as a loading control. A representative Western blot from more than 3 independent experiments is shown.

The inhibitory factor produced by B. thetaiotaomicron could be affecting S. flexneri virulence gene expression directly or indirectly by affecting expression of upstream regulators. In S. flexneri, VirF is the master virulence transcriptional regulator, which activates expression of a second regulatory gene, *virB*. VirB regulates other virulence genes, including the *ipa* genes. Additionally, *icsA* is directly regulated by VirF but not by VirB (6–8) (Fig. 3A). Since both IcsA and Ipa protein levels were reduced, this suggested that B. thetaiotaomicron CM represses *virF*. To test whether B. thetaiotaomicron CM affects S. flexneri virulence gene expression, the relative virulence gene expression level of S. flexneri grown in the presence or absence of B. thetaiotaomicron CM was determined by quantitative reverse transcription-PCR (RT-qPCR). *virB* and *icsA* expression was repressed 5.7- and 6.4-fold, respectively, in the presence of B. thetaiotaomicron CM. Additionally, VirB’s downstream targets *ipaB* and *ipaC* were repressed 8.8- and 9.0-fold (Fig. 3B), consistent with B. thetaiotaomicron CM repression of *virB*. Surprisingly, *virF* levels were unchanged. This indicated either that B. thetaiotaomicron CM repression of its two targets, *virB* and *icsA*, was independent of VirF or that VirF levels were reduced posttranscriptionally. To verify that the effect of B. thetaiotaomicron CM on S. flexneri virulence gene expression was specific to B. thetaiotaomicron and not due to general effects of nutrient depletion on S. flexneri, CM from E. coli MG1655 was collected and its effect on S. flexneri virulence gene expression was determined. E. coli CM did not affect the expression of any S. flexneri virulence genes tested (Fig. S2), indicating that these effects are not simply due to nutrient depletion. To determine if B. thetaiotaomicron CM regulates VirF posttranscriptionally, an S tag was fused in frame to the 3′ end of *virF*, and the tagged gene was expressed from its native promoter on a plasmid. Western blot analysis of S-tagged VirF showed that VirF protein levels were reduced in B. thetaiotaomicron CM relative to BHIS (Fig. 3C). These data, showing that B. thetaiotaomicron CM reduces VirF S-tag levels (Fig. 3C), but not *virF* gene expression (Fig. 3B), suggest that B. thetaiotaomicron CM affects VirF levels posttranscriptionally.

10.1128/mbio.02360-22.2 FIG S2 E. coli CM does not affect S. flexneri virulence gene expression. Relative virulence gene expression of S. flexneri grown to log phase in either BHIS or one-half E. coli CM was measured by RT-qPCR. The E. coli CM was tested in parallel with the B. thetaiotaomicron CM shown in Fig. 3B, and the BHIS control values are the same as shown in that figure. *C_T_* values were normalized to the mean for two endogenous controls, *gyrA* and *secA*. *P* values were determined from the Δ*C_T_* values of three biological replicates using a two-tailed Student *t* test with Holm-Šidák correction for multiple comparisons. Error bars indicate standard deviation. Download FIG S2, DOCX file, 0.03 MB. Copyright © 2022 Xerri and Payne. 2022 Xerri and Payne. https://creativecommons.org/licenses/by/4.0/ This content is distributed under the terms of the Creative Commons Attribution 4.0 International license.

### B. thetaiotaomicron inhibitory factor is not a secreted metabolite or protein.

**FIG 4:**
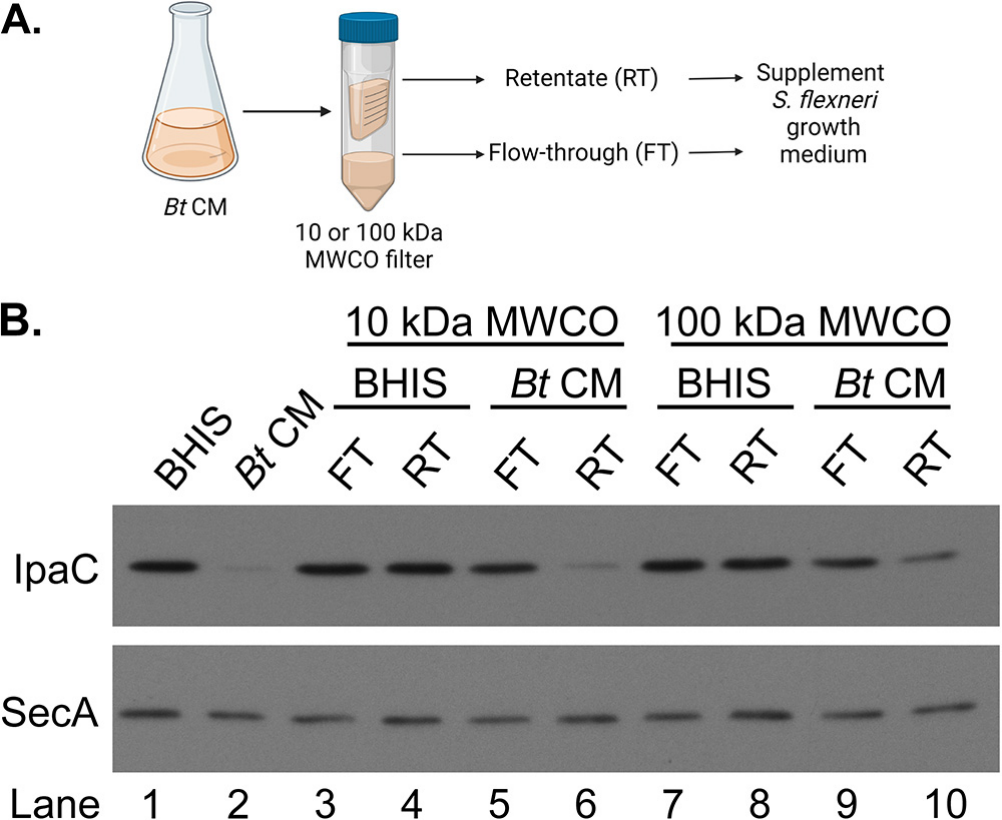
Fractionation of B. thetaiotaomicron CM shows that the active fraction is not a small molecule. (A) B. thetaiotaomicron CM was fractionated by size using either a 10-kDa-molecular-weight-cutoff (MWCO) filter or a 100-kDa-MWCO filter. The retentate fraction (RT) or the flowthrough (FT) was added to S. flexneri growth medium. As a negative control, BHIS was subjected to the same fractionation. Illustration created with BioRender.com. (B) Growth in the presence of the retentate fraction of B. thetaiotaomicron CM from either a 10-kDa or a 100-kDa filter inhibits S. flexneri IpaC expression. Anti-SecA was used as a loading control. A representative Western blot from 3 independent fractionation experiments is shown.

To characterize the nature of the B. thetaiotaomicron secreted inhibitory factor, we looked for evidence that the inhibitory factor was either a secreted metabolite or a secreted protein. Bacteria of the gut microbiota, including *Bacteroides* species, are known to excrete a variety of short-chain fatty acids (SCFAs) (37, 38), and enteric pathogens have been shown to modulate their virulence genes in response to exogenous SCFAs (39–41). To determine whether S. flexneri was responding to a secreted metabolite, such as an SCFA, the small molecules (<10 kDa) were separated from proteins and other large molecules using a protein concentrator (Fig. 4A). The inhibitory activity of fractionated B. thetaiotaomicron CM was associated with the retentate; the small molecules in the flowthrough had no effect on the virulence protein levels (Fig. 4B, lanes 5 and 6). We repeated the fractionation of B. thetaiotaomicron CM with a 100-kDa-molecular-weight-cutoff filter. Similar to what was observed with the 10-kDa-cutoff filter, the majority of the inhibitory activity remained in the retentate (Fig. 4B, lanes 9 and 10), suggesting that the active factor was not a small molecule. As expected, fractionating BHIS with the protein concentrators had no effect on IpaC expression relative to unfractionated BHIS (Fig. 4B, lanes 3 and 4 and lanes 7 and 8). The lack of effect of the flowthrough on the level of S. flexneri virulence proteins indicates that the inhibition is due to the presence of a high-molecular-weight factor and further demonstrates that it is not a result of depletion of a growth factor from the conditioned medium.

**FIG 5:**
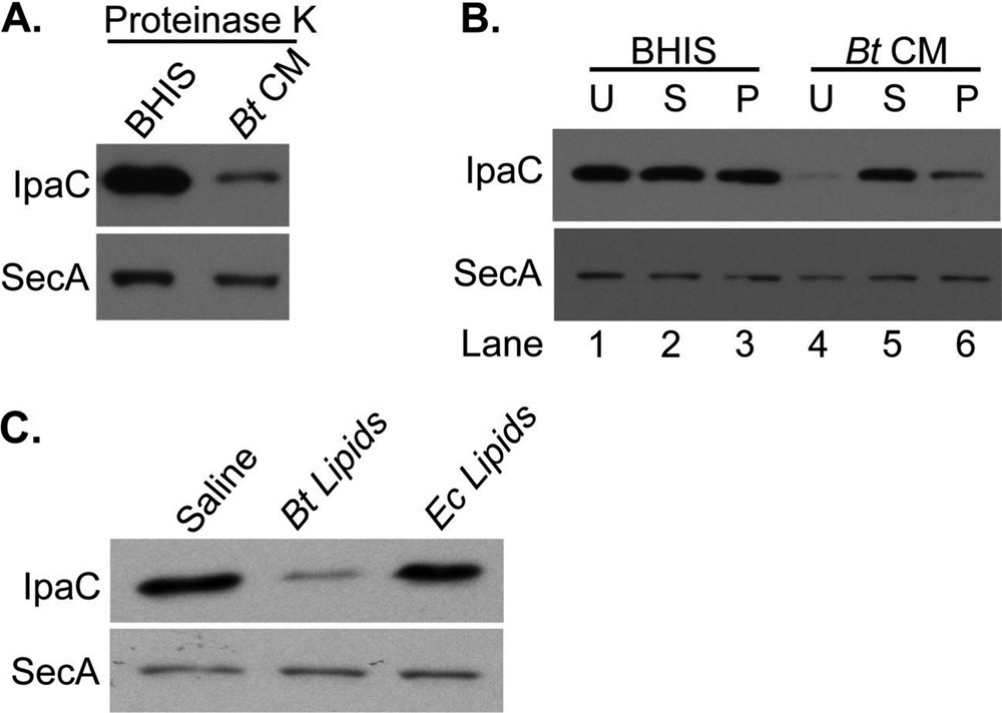
Active component of B. thetaiotaomicron CM is lipid associated and proteinase K resistant. (A) To degrade proteins, BHIS and B. thetaiotaomicron CM were treated with 50 μg/mL of proteinase K. Each was then mixed 1:1 with untreated BHIS. Total proteins were collected from S. flexneri grown to mid-log phase in either proteinase K-treated BHIS or proteinase K-treated B. thetaiotaomicron CM and used for an anti-IpaC Western blot assay. (B) BHIS and B. thetaiotaomicron CM were ultracentrifuged to separate the water-soluble supernatant from the insoluble pellet. The supernatant was mixed 1:1 with BHIS, while the pellet was resuspended in BHIS. Total proteins were collected from S. flexneri grown to log phase in unfractionated medium (U) or BHIS supplemented with either the supernatant (S) or pellet (P) fractions and used for an anti-IpaC Western blot assay. (C) S. flexneri was grown in the presence of B. thetaiotaomicron lipids, E. coli (*Ec*) lipids, or an equal volume of saline. The effect of these lipids on S. flexneri IpaC levels was determined by Western blotting. For all three panels, anti-SecA was used as a loading control. Each fractionation or isolation and its corresponding Western blot assay were performed at least 3 independent times.

To assess whether the inhibitor was a protein, B. thetaiotaomicron CM was treated with proteinase K. However, proteinase K treatment of B. thetaiotaomicron CM did not eliminate its ability to inhibit S. flexneri IpaC production (Fig. 5A), suggesting the active component was not a protein. It was possible that the B. thetaiotaomicron secreted factor was a soluble metabolite that nonspecifically stuck to the protein concentrator during fractionation and was recovered in the retentate fraction. To rule this out, we fractionated B. thetaiotaomicron CM by ultracentrifugation. This separated the supernatant, containing soluble molecules, from a pellet, containing the lipids and other insoluble components of the CM. These two fractions were then tested individually for their effect on S. flexneri, where it was observed that S. flexneri grown in medium containing the resuspended pellet had reduced IpaC expression (Fig. 5B, lane 6), while S. flexneri grown in medium supplemented with the supernatant (Fig. 5B, lane 5) had IpaC levels comparable to those of the BHIS control (lane 1). This indicated that the active component in B. thetaiotaomicron-conditioned medium was insoluble in aqueous solution and likely to be lipid associated. To ensure that the inhibitory factor being ultracentrifuged out of B. thetaiotaomicron CM was specific to the CM, BHIS was fractionated in the same way. Neither the supernatant nor the pellet isolated from BHIS had any effect on S. flexneri IpaC levels (Fig. 5B, lanes 1 to 3).

### B. thetaiotaomicron lipids and OMVs inhibit S. flexneri virulence factors.

To directly determine whether the inhibition of S. flexneri virulence factor production was due to the presence of B. thetaiotaomicron lipids in the culture supernatant, we extracted the total lipids from a pellet of stationary-phase B. thetaiotaomicron and grew S. flexneri in the presence of these lipids. Growth in the presence of total B. thetaiotaomicron lipids repressed IpaC expression (Fig. 5C). This effect was specific to B. thetaiotaomicron lipids, since an equivalent amount of lipids from E. coli had no effect (Fig. 5C).

**FIG 6:**
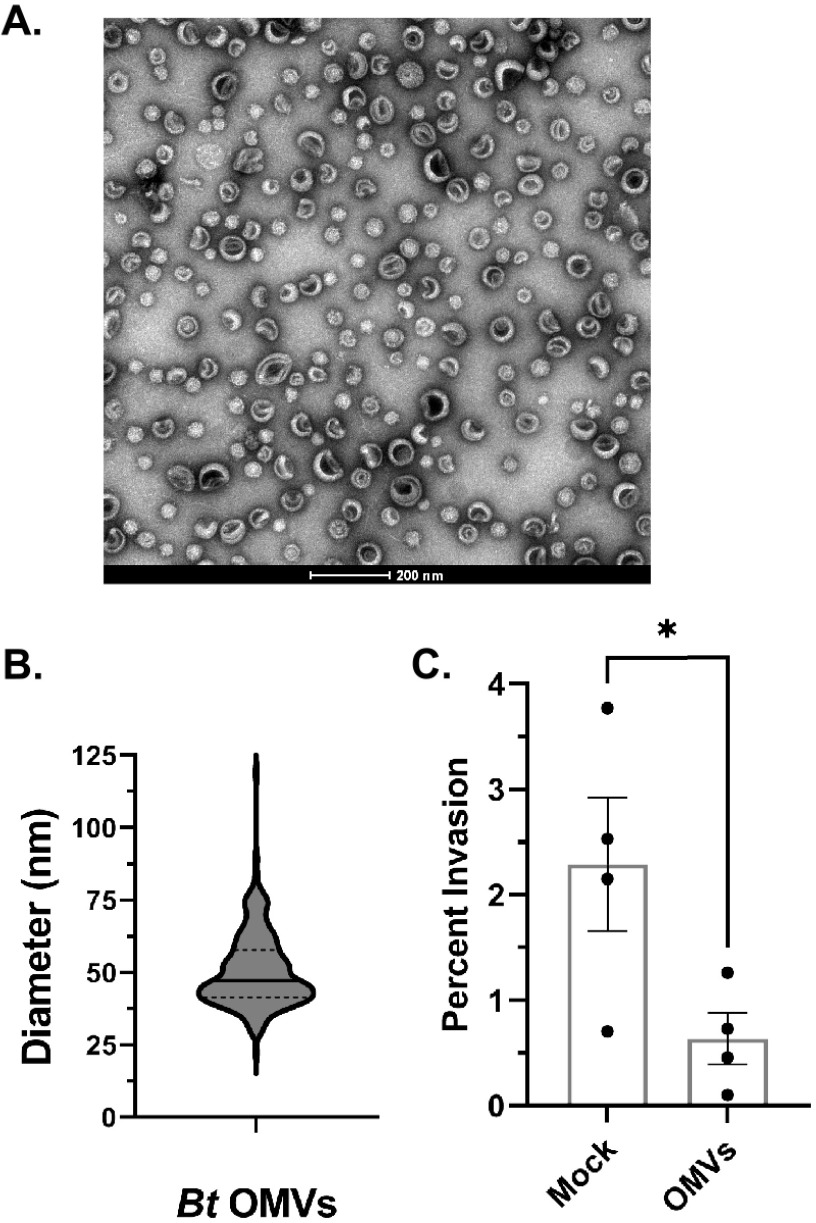
B. thetaiotaomicron CM contains OMVs that inhibit S. flexneri invasion. (A) Extracellular vesicles were concentrated from B. thetaiotaomicron CM, purified on a density gradient, and counterstained with 2% uranyl acetate for visualization by transmission electron microscopy. A representative image taken at ×60,000 magnification is shown. Bar, 200 nm. (B) The diameters of 400 OMVs were measured using ImageJ (88). (C) A gentamicin protection assay was used to determine the invasion rate of S. flexneri grown in the presence of 20 μg/mL of B. thetaiotaomicron OMVs or an equal volume of mock extract. *P* value was determined by a two-tailed, unpaired *t* test from 4 biological replicates (*, *P* < 0.05). Error bars indicate standard error of the mean.

Gram-negative bacteria, including B. thetaiotaomicron, are known to produce outer membrane vesicles (OMVs). OMVs have been shown to be involved in cell-to-cell communication (42). Because the size and lipid nature of the inhibitory factor were consistent with OMVs, we purified OMVs from the B. thetaiotaomicron CM for analysis. Transmission electron microscopy (TEM) confirmed the presence of small spherical structures (Fig. 6A) ranging from 20 nm to 100 nm in diameter, with an average diameter of 51 nm (Fig. 6B). These structures were similar in both size and appearance to OMVs previously isolated from other *Bacteroides* species (43). Additionally, we validated that the vesicles isolated from B. thetaiotaomicron CM were OMVs by utilizing B. thetaiotaomicron strains expressing tagged proteins known to localize to either the outer membrane (OM) or OMVs extracted from these strains. Consistent with the results of Valguarnera et al. (44), BT_1488 was localized to the isolated extracellular vesicles, while BT_0418 was found in the OM and not in the extracellular vesicles (Fig. S3). This indicates that the purified fraction contained B. thetaiotaomicron OMVs and was not significantly contaminated with cellular membranes.

10.1128/mbio.02360-22.3 FIG S3 B. thetaiotaomicron OMVs have the expected protein profile: BT_0418 is localized to the outer membrane, while BT_1488 is localized to outer membrane vesicles. Total membrane (TM), inner membrane (IM), outer membrane (OM), or outer membrane vesicles (OMV) were extracted from B. thetaiotaomicron::pFD340/BT_0418-6xHis and B. thetaiotaomicron::pFD340/BT_1488-6xHis strains. Membrane preparations were normalized using a DC protein assay, and 10 μg of sample was loaded per well for an anti-His Western blotting. Download FIG S3, DOCX file, 0.5 MB. Copyright © 2022 Xerri and Payne. 2022 Xerri and Payne. https://creativecommons.org/licenses/by/4.0/ This content is distributed under the terms of the Creative Commons Attribution 4.0 International license.

**FIG 7:**
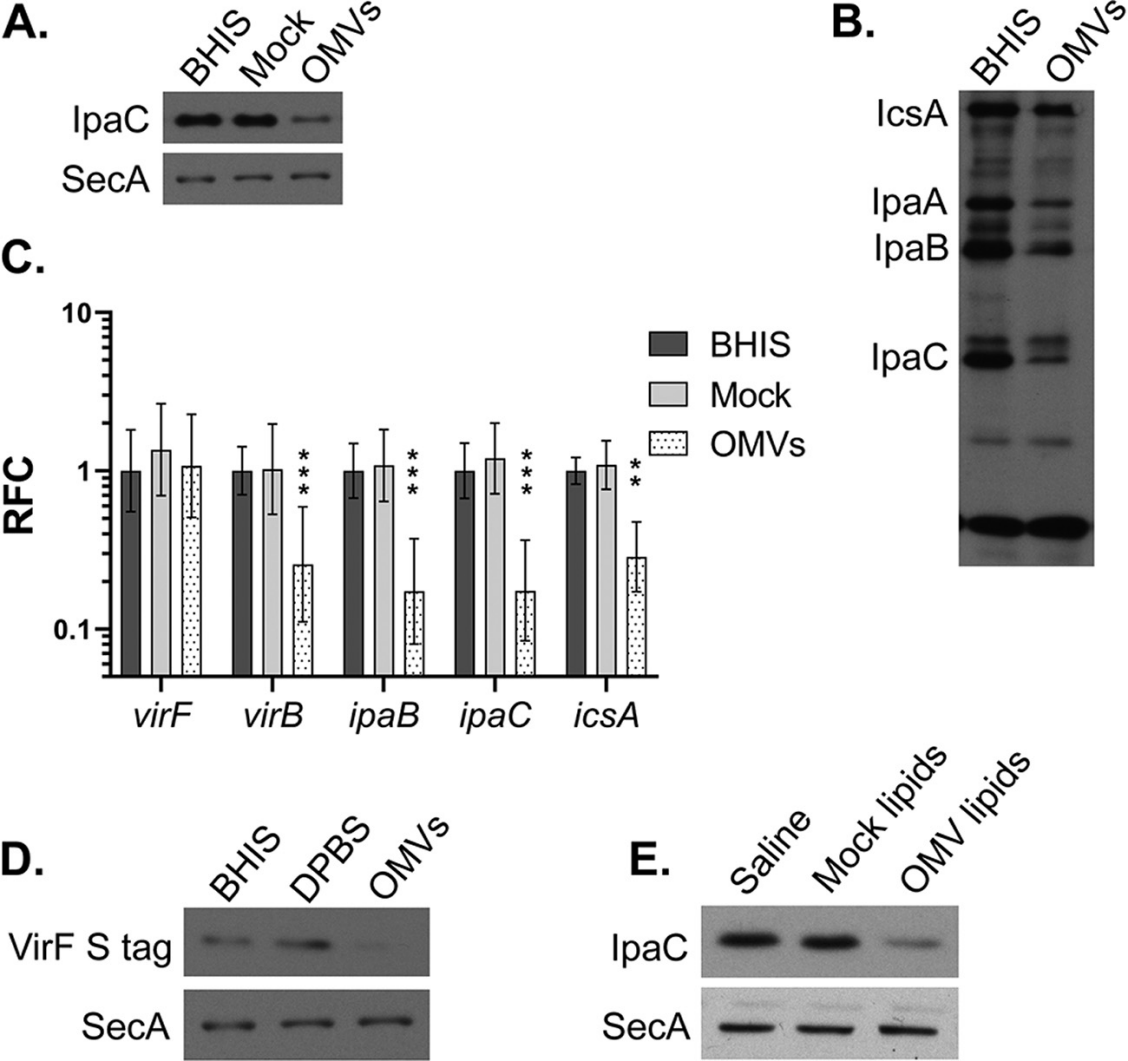
B. thetaiotaomicron OMVs repress S. flexneri virulence gene expression. (A) An anti-IpaC Western blot assay was performed on proteins collected from S. flexneri that was either untreated, treated with mock extract, or treated with 20 μg/mL B. thetaiotaomicron OMVs and grown to log phase. Anti-SecA was used as a loading control. (B) Proteins were collected from untreated S. flexneri and S. flexneri treated with 20 μg/mL of B. thetaiotaomicron OMVs. A Western blot assay using monkey anti*-Shigella* convalescent-phase antiserum was performed to look at the relative protein levels of a panel of virulence factors. (C) Relative fold change (RFC) of virulence gene expression of S. flexneri treated with either mock extract or 20 μg/mL of B. thetaiotaomicron OMVs compared to untreated S. flexneri was measured by RT-qPCR. *C_T_* values were normalized to the mean for two endogenous controls, *gyrA* and *secA*. *P* values were determined from the Δ*C_T_* values of three biological replicates using two-way analysis of variance with Dunnett’s posttest for multiple comparisons of gene expression in treated samples to that in the untreated samples (*, *P* < 0.05; **, *P* < 0.01; ***, *P* < 0.001). Error bars indicate standard deviation. (D) An anti-S-peptide Western blot assay was performed on proteins collected from S. flexneri pWKS30::*virF*_*S-tag* that was either untreated, treated with Dulbecco’s phosphate-buffered saline supplemented with salts (DPBS), or treated with 40 μg/mL B. thetaiotaomicron OMVs and grown to log phase. Anti-SecA was used as a loading control. (E) S. flexneri was grown to log phase in the presence of saline, lipids extracted from B. thetaiotaomicron OMVs, or an equal volume of mock lipids. An anti-IpaC Western blot assay was performed. Anti-SecA was used as the loading control. Each Western blot in this figure is representative of at least 3 independent replicates, except for panel B, which is representative of 2 independent replicates.

To determine whether B. thetaiotaomicron OMVs are the factor responsible for repressing S. flexneri virulence in B. thetaiotaomicron CM, the invasion rate of S. flexneri grown in the presence of B. thetaiotaomicron OMVs was measured. The amount of OMVs added was normalized to protein levels in the purified vesicles and is consistent with concentrations used in previous studies (45, 46). We observed that when treated with B. thetaiotaomicron OMVs, S. flexneri was less invasive than S. flexneri treated with an extract of uninoculated BHIS (mock extract) (Fig. 6C). Similar to the results with B. thetaiotaomicron CM, B. thetaiotaomicron OMVs inhibited expression of S. flexneri IpaC (Fig. 7A) and the virulence factors IcsA, IpaA, and IpaB (Fig. 7B). As determined by RT-qPCR, this reduction in protein levels was associated with reduced expression of the virulence genes *virB*, *icsA*, *ipaB*, and *ipaC*. Relative to growth in BHIS alone, S. flexneri grown in the presence of B. thetaiotaomicron OMVs had reductions in expression of 3.9-fold for *virB*, 3.5-fold for *icsA*, 5.8-fold for *ipaB*, and 5.7-fold for *ipaC*. Also consistent with the results from B. thetaiotaomicron CM treatment, v*irF* gene expression was unchanged by growth in the presence of the B. thetaiotaomicron OMVs (Fig. 7C), but protein levels of VirF were reduced (Fig. 7D). Thus, the gene and protein expression patterns observed in B. thetaiotaomicron CM were recapitulated by B. thetaiotaomicron OMVs. The level of repression of the virulence genes by the vesicles is somewhat less than that observed with the conditioned medium. This may reflect differences in the amount of the inhibitory factor between the purified vesicles and the crude conditioned medium, or there may be an additive effect of the vesicles and some other component in the conditioned medium.

### B. thetaiotaomicron OMV lipids inhibit IpaC.

Since B. thetaiotaomicron lipids and OMVs both repress S. flexneri IpaC levels, we wanted to determine whether B. thetaiotaomicron OMV lipids alone were sufficient to inhibit S. flexneri IpaC expression. Lipids extracted from purified B. thetaiotaomicron OMVs repressed S. flexneri IpaC levels (Fig. 7E), indicating that the inhibitory effect of the OMVs is due to the OMV lipids themselves and not to the presence of an unknown cargo contained in the B. thetaiotaomicron OMVs. Performing the same OMV and lipid extraction starting with BHIS (mock lipids), and growing S. flexneri in the presence of this extract had no effect on IpaC levels (Fig. 7E), demonstrating that the extracted lipids that inhibit S. flexneri virulence protein levels are specific to B. thetaiotaomicron OMVs and not present in the uninoculated BHIS medium.

### B. thetaiotaomicron OMVs directly interact with S. flexneri.

**FIG 8:**
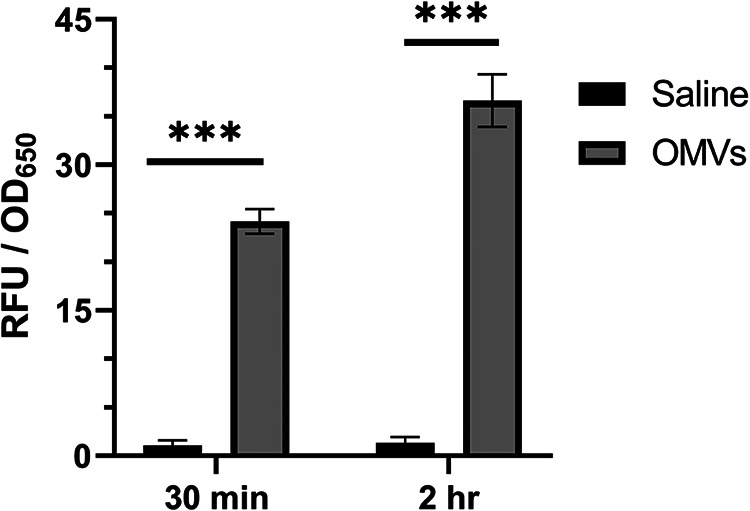
B. thetaiotaomicron OMVs fuse to S. flexneri. Saline or B. thetaiotaomicron OMVs were stained with 5 μg/mL of the fluorescent dye FM4-64 FX, washed extensively, and incubated with S. flexneri for 2 h at room temperature. At time points of 30 min and 2 h, S. flexneri was fixed with 4% paraformaldehyde (PFA), and its fluorescence was measured on a plate reader (Ex/Em of 510/640 nm). *P* values were determined from three biological replicates using a two-tailed Student *t* test with the Holm-Šidák correction for multiple comparisons. (***, *P* < 0.001). Error bars indicate standard deviation.

Because the effect of B. thetaiotaomicron-produced OMVs on S. flexneri invasion is due to the lipids in B. thetaiotaomicron OMVs, we hypothesized that the OMVs were fusing with S. flexneri’s membrane and that the presence of B. thetaiotaomicron lipids in the membrane of S. flexneri could initiate the observed changes in S. flexneri virulence protein levels. To test whether the B. thetaiotaomicron vesicles directly contact S. flexneri, B. thetaiotaomicron OMVs were stained with the lipophilic fluorescent dye FM4-64 FX and washed extensively to remove unbound dye. The stained B. thetaiotaomicron OMVs were then coincubated with S. flexneri, and the amount of fluorescence associated with the S. flexneri cells was determined over time. After 30 min of coincubation, S. flexneri exhibited a fluorescent signal of 24.2 relative fluorescent units (RFU)/optical density at 650 nm (OD_650_), and after 2 h of coincubation, the fluorescence of S. flexneri increased to 36.6 RFU/OD_650_ (Fig. 8). This suggested that the fluorescently labeled OMVs fused with S. flexneri. Since free FM4-64 FX can directly bind to S. flexneri, we controlled for free dye carryover by performing the same staining and washing procedure on saline that did not contain OMVs. After 30 min and 2 h of coincubation with the stained and washed control saline, S. flexneri exhibited fluorescence of only 1.1 and 1.3 RFU/OD_650_, respectively. This ruled out dye carryover from the staining and washing procedures as the cause of S. flexneri fluorescence. To further control for release of dye from the vesicles during coincubation, labeled vesicles were soaked in buffer for the same amount of time used in the transfer experiment. After soaking, the vesicles were removed by ultracentrifugation, and S. flexneri was incubated with either the soaked OMVs or the buffer in which the OMVs had been soaked. While some fluorescence was measured in S. flexneri that was resuspended in the soaking buffer, the amount of fluorescence was over 10 times lower than that of S. flexneri that was incubated with the soaked OMVs. Additionally, the fluorescence of S. flexneri that was coincubated with soaked OMVs was indistinguishable from that of S. flexneri that was coincubated with unsoaked OMVs (Fig. S4). Together, this suggests that passive transfer of dye from the OMVs to S. flexneri via diffusion of dye away from the stained vesicles is only a minor contributor to S. flexneri fluorescence and indicates that fusion or direct contact between the OMVs and S. flexneri is likely occurring.

10.1128/mbio.02360-22.4 FIG S4 Direct contact between B. thetaiotaomicron OMVs and S. flexneri is required for dye transfer. B. thetaiotaomicron OMVs were stained with 5 μg/mL of the fluorescent dye FM4-64 FX, washed extensively, soaked in saline for 30 min, and then separated out of their soaking solution. S. flexneri was incubated in the OMV soaking solution, the presence of soaked OMVs, or the presence of unsoaked stained OMVs. At a time point of 2 h, S. flexneri fluorescence was measured on a plate reader (Ex/Em of 510/640 nm). *P* values were determined from three biological replicates using a one-way analysis of variance with Dunnett’s test for multiple comparisons (*, *P* < 0.05). Error bars indicate standard error. Download FIG S4, DOCX file, 0.03 MB. Copyright © 2022 Xerri and Payne. 2022 Xerri and Payne. https://creativecommons.org/licenses/by/4.0/ This content is distributed under the terms of the Creative Commons Attribution 4.0 International license.

## DISCUSSION

Despite the fact that S. flexneri must interact with the gut microbiota before establishing infection in the colon, the impact of these interactions on S. flexneri pathogenesis remains poorly understood. In this study, we show that the common gut microbe B. thetaiotaomicron produces OMVs that repress S. flexneri virulence gene expression and inhibit S. flexneri invasion.

These results have implications in the broader context of S. flexneri pathogenesis. First, it is possible that gut microbiotas enriched for B. thetaiotaomicron have a protective effect, making the people that harbor these microbiotas more resistant to S. flexneri infection. In humans, *Bacteroides* is often the most abundant genus in the gut microbiota. However, it also the most variable, ranging from comprising the majority of gut microbes in some people to comprising only a small minority in others (22, 24). Our data show that the inhibitory effect of B. thetaiotaomicron on S. flexneri virulence protein levels is dose dependent, with a higher proportion of B. thetaiotaomicron CM leading to a stronger reduction in the T3SS protein IpaC (Fig. 2A), and that the presence of OMVs in B. thetaiotaomicron CM is responsible for its inhibitory effect on S. flexneri invasion (Fig. 6). We speculate that if the abundance of B. thetaiotaomicron in the gut microbiota were to correlate with the accumulation of more B. thetaiotaomicron OMVs in the lumen of the colon, then it is conceivable that individuals with higher proportions of B. thetaiotaomicron could be given some degree of protection from *Shigella* infection. However, existing data suggesting that B. thetaiotaomicron plays a protective role in S. flexneri infection are scarce. One study examined the composition of the gut microbiota in children in low-income countries in the presence or absence of *Shigella* (16). These data show that the relative abundance of *Bacteroides* is lower in children who are colonized by *Shigella* and have diarrhea than in children who are colonized by *Shigella* but do not have diarrhea. While this correlation between *Bacteroides* proportion and disease state is consistent with the possibility that *Bacteroides* plays a protective role in *Shigella* infection, it is worth noting that a similar decrease in abundance of *Bacteroides* was observed in children who had diarrhea relative to children who did not have diarrhea, even when these children did not have *Shigella*. Thus, it is difficult to parse out whether these effects are due to interactions between *Bacteroides* and *Shigella*, or whether they are simply due to effects of diarrhea on gut microbiota composition. More research is needed to determine whether the abundance of certain species in the gut microbiota can affect susceptibility to S. flexneri infection.

Alternatively, it is possible that, rather than repressing S. flexneri virulence, B. thetaiotaomicron functions to help coordinate S. flexneri invasion. The low infectious dose of *Shigella* (47) suggests that its invasion process is both highly coordinated and efficient. Furthermore, a number of cues, including temperature (48), pH (49), oxygen tension (50), bile salts (51), and osmolarity (52), which help coordinate S. flexneri invasion have been identified. Perhaps B. thetaiotaomicron-produced OMVs are functioning as another cue that prevents S. flexneri from prematurely expressing its T3SS in the lumen of the colon, before other cues closer to the colonic epithelium trigger T3SS expression at the appropriate time. This paradigm, where members of the gut microbiota secrete cues that are sensed by enteric pathogens and used to coordinate infection, has been reported in other enteric pathogens (33, 35).

The inhibitory effect of B. thetaiotaomicron CM on S. flexneri virulence is due to the presence of B. thetaiotaomicron OMVs (Fig. 6 and 7). OMVs derived from commensal bacteria are known to modulate the intestinal immune response, deliver molecules that protect mice from colitis, and function in interkingdom signaling between bacteria and intestinal cells, while OMVs from pathogenic bacteria have been shown to deliver virulence factors to intestinal cells (53–58). In the context of interbacterial interaction, OMVs have been observed to affect population and community dynamics by functioning in both intraspecies and interspecies signaling (42, 59, 60). In this study, we add to the variety of ways that OMVs are known to function in interspecies interaction by showing that OMVs secreted by one species of bacteria can modulate the virulence gene expression of another species. Additionally, by showing that gut microbiota-derived OMVs can modulate the invasiveness of an enteric pathogen, we have identified a possible new function for bacterial OMVs in the colon. Whether this inhibitory effect is specific to B. thetaiotaomicron OMVs and S. flexneri virulence genes or is more generalizable to other combinations of gut microbes and enteric pathogens is unknown.

The mechanism by which B. thetaiotaomicron OMVs repress S. flexneri virulence gene expression remains to be determined. The interaction between the bacteria involves direct contact between B. thetaiotaomicron OMVs and S. flexneri, the majority of which occurs within the first 30 min of coincubation (Fig. 8). This suggests that the vesicles are fusing with the S. flexneri cells. Additionally, incubation with B. thetaiotaomicron OMVs leads to a decrease in protein levels of the S. flexneri master virulence regulator, VirF (Fig. 7D). The link between OMV fusion and repression of VirF remains uncharacterized; however, it is possible that the fusion of lipids contained in B. thetaiotaomicron OMVs into the membrane of S. flexneri triggers stress responses. Virulence gene expression in S. flexneri and other enteric pathogens is known to be closely intertwined with bacterial stress responses (61–66). B. thetaiotaomicron OMVs fusing to S. flexneri may activate envelope stress response two-component systems, and then these directly or indirectly transduce the signal of B. thetaiotaomicron OMV fusion from the outer membrane of S. flexneri to VirF. While B. thetaiotaomicron OMV-induced repression of VirF protein was observed, no change in *virF* RNA was detected (Fig. 7C). This suggests posttranscriptional regulation or effects on the stability of VirF. To date, two examples of posttranscriptional regulation of VirF have been described. Specifically, deletion of two genes involved in tRNA modification, *tgt* and *miaA*, has been shown to decrease the translation efficiency of *virF* despite having no effect on its transcription (7, 67, 68). Future studies will be aimed at determining how OMV fusion leads to the observed decrease in VirF.

Our data suggest that the particular component of B. thetaiotaomicron OMVs that causes the repression of S. flexneri virulence gene expression is a lipid. Total lipid extracts from both B. thetaiotaomicron stationary-phase cell pellet and B. thetaiotaomicron OMVs repress S. flexneri IpaC protein expression, while an equivalent amount of lipids from E. coli does not (Fig. 5C). The particular B. thetaiotaomicron lipid or lipids that are responsible for this effect remain unknown. Since E. coli lipids do not induce the same effect, it does not appear to be a general lipid effect that reduces IpaC levels. Furthermore, this specificity for B. thetaiotaomicron lipids indicates that the responsible inhibitory lipid(s) is likely enriched in B. thetaiotaomicron relative to E. coli, or absent from E. coli altogether. B. thetaiotaomicron membrane sphingolipids are a possible candidate. Unlike E. coli and almost all other bacteria, members of the phylum *Bacteroidota* (including B. thetaiotaomicron) contain membrane sphingolipids, which comprise over 50% of the total lipids in B. thetaiotaomicron OMVs (69–71). Sphingolipids of both mammalian and bacterial origin can be found in the colon, where B. thetaiotaomicron sphingolipids are known to promote intestinal homeostasis (72, 73). In the environment, the bacterium Algoriphagus machipongonensis induces multicellular rosette formation of the choanoflagellate Salpingoeca rosetta by releasing OMVs that fuse to *S. rosetta*. The active signals contained in the OMVs that induce *S. rosetta* rosette formation are sulfonolipids, a structural analogue of sphingolipids (74, 75). A similar signaling mechanism may be occurring in S. flexneri, whereby B. thetaiotaomicron sphingolipids, released as part of OMVs, fuse to S. flexneri and cause the observed repression of virulence gene expression.

## MATERIALS AND METHODS

### Bacterial strains and growth conditions.

Bacterial strains and plasmids used in this study can be found in Table S1 in the supplemental material. S. flexneri and E. coli strains were maintained at −80°C in tryptic soy broth (TSB) containing 20% (vol/vol) glycerol. S. flexneri was grown aerobically at 37°C on TSB agar with 0.01% Congo red dye. Overnight cultures in TSB were subcultured 1:100 into the indicated growth medium and grown aerobically (200 rpm, 37°C). E. coli strains were grown on Luria-Bertani (LB) agar (1% Tryptone, 0.5% yeast extract, 1% NaCl, and 1% agar) and in LB broth (1% Tryptone, 0.5% yeast extract, 1% NaCl) under the same growth conditions as S. flexneri.

10.1128/mbio.02360-22.5 TABLE S1 Strains and plasmids used in this study. Download Table S1, DOCX file, 0.02 MB. Copyright © 2022 Xerri and Payne. 2022 Xerri and Payne. https://creativecommons.org/licenses/by/4.0/ This content is distributed under the terms of the Creative Commons Attribution 4.0 International license.

B. thetaiotaomicron strains were maintained at −80°C, in brain heart infusion (BHI; Bacto) containing 5 mg/L hemin and 20% (vol/vol) glycerol. B. thetaiotaomicron was grown on BHI agar supplemented with yeast extract (0.5% [wt/vol]), sodium bicarbonate (0.2% [wt/vol]), 5 mg/L hemin, and l-cysteine (free base, 0.1% [wt/vol]). For liquid culture, B. thetaiotaomicron was grown in supplemented brain heart infusion (BHIS) containing 37 g/L brain heart infusion (Bacto), yeast extract (0.5% [wt/vol]), 5 mg/L hemin, l-cysteine (free base, 0.1% [wt/vol]), and 50 mM HEPES sodium salt (pH 7.5). B. thetaiotaomicron was cultured in an anaerobic chamber (Coy) using an atmosphere of 85% N_2_, 10% CO_2_, 5% H_2_ at 37°C. Antibiotics were used at the indicated concentrations: gentamicin (20 μg/mL) and erythromycin (25 μg/mL).

### Construction of plasmids.

C-terminally S-tagged (76) VirF (VirF_S-tag) was constructed using the primers indicated in Table S2. The promoter region and coding sequence of *virF* were amplified with the primers from the virulence plasmid of S. flexneri 2457T along with the S tag, which was included in the sequence of the reverse primer. The region directly downstream of *virF* on the virulence plasmid was amplified as well. These two pieces were attached using splicing by overlap extension PCR (77) and then ligated into the EcoRI and SalI sites of the low-copy-number vector pWKS30 (78).

10.1128/mbio.02360-22.6 TABLE S2 Primers used in this study. Download Table S2, DOCX file, 0.02 MB. Copyright © 2022 Xerri and Payne. 2022 Xerri and Payne. https://creativecommons.org/licenses/by/4.0/ This content is distributed under the terms of the Creative Commons Attribution 4.0 International license.

### Cell culture media and growth conditions.

Minimal essential medium (MEM; Gibco) containing heat-inactivated fetal bovine serum (Gibco; 10% [vol/vol]), tryptone phosphate broth (Bacto; 10% [wt/vol]), 1× nonessential amino acids (Gibco), and 2 mM glutamine was used to grow Henle cells (intestine 407; ATCC CCL-6). Henle cells were incubated at 37°C with 5% CO_2_.

### B. thetaiotaomicron cell-free CM.

To generate B. thetaiotaomicron-conditioned medium (CM), B. thetaiotaomicron was grown anaerobically on BHI agar plus gentamicin. BHIS was inoculated with a single colony of B. thetaiotaomicron and grown anaerobically for 40 h to late stationary phase. The stationary-phase culture was centrifuged at 13,000 × *g* for 10 min. The supernatant was filter sterilized using a 0.22-μm polyether sulfone (PES) filter, pH adjusted to 6.8 with 1 M NaOH, and then passed through a second 0.22-μm filter.

### Virulence assays.

S. flexneri invasion was measured by a gentamicin protection assay (79). S. flexneri was grown to late log phase in the indicated growth medium. Approximately 10^8^ S. flexneri CFU (multiplicity of infection of ~100) were added to a confluent monolayer of Henle cells in a 35-mm, 6-well polystyrene plate (Corning) and centrifuged at 1,000 × *g* for 10 min. The plate was incubated for 30 min at 37°C and 5% CO_2_, after which each well was washed 4 times with phosphate-buffered saline (PBS-D) (1.98 g/L KCl, 8 g/L NaCl, 0.02 g/L KH_2_PO_4_, 1.39 g/L K_2_HPO_4_), filled with MEM containing 20 μg/mL gentamicin, and incubated for another hour. The wells were then washed twice more with PBS-D and lysed at room temperature for 10 min with 0.1% Triton X-100 to recover intracellular bacteria. Tenfold serial dilutions of both the input and output bacteria were plated, and invasion efficiency for each sample was calculated as percent invasion (output CFU/input CFU). For plaque assays, S. flexneri was grown to log phase in LB, and 5 × 10^4^ bacteria were added to a confluent layer of Henle cells in a 6-well polystyrene plate and centrifuged at 1,000 × *g* for 10 min. The plate was incubated for 30 min, after which each well was washed 4 times with PBS-D, and the medium was replaced with MEM containing 0.45% glucose and gentamicin for 24 h. After 24 h, the medium was replaced with MEM containing only gentamicin, and the plate was incubated for 48 h more. The wells were washed with PBS-D, fixed with 80% methanol for 5 min, and then stained with 0.5% crystal violet for visualization.

### SDS-PAGE and immunoblotting.

S. flexneri was grown to late log phase in the indicated growth medium, centrifuged, resuspended in Laemmli SDS sample buffer (5% β-mercaptoethanol, 3% [wt/vol] SDS, 10% glycerol, 0.02% [wt/vol] bromophenol blue, 63 mM Tris-Cl [pH 6.8]) (80) at a concentration of approximately 2 × 10^9^ CFU/mL, and boiled for 5 min. To isolate secreted proteins, Halt protease inhibitor (ThermoFisher) was added to S. flexneri supernatant, which was then concentrated 15-fold using a 10-kDa-molecular-weight cutoff filter (Amicon). Concentrated supernatant was normalized to the OD_650_ of the bacterial culture from which it was collected and then resuspended in 4× Laemmli SDS sample buffer.

Samples were run on a 10% SDS-PAGE gel and then either stained with Coomassie brilliant blue or transferred to a 0.45-μm-pore-size nitrocellulose membrane (GE Healthcare) for immunoblotting with mouse monoclonal anti-S-peptide antibody (Thermo Fisher) diluted 1:1,000, mouse monoclonal anti-6×His (Thermo Fisher) diluted 1:1,000, rabbit polyclonal anti-SecA antibody (Donald Oliver, Wesleyan University) diluted 1:10,000, mouse monoclonal anti-IpaC (Edwin Oaks, Walter Reed Army Institute of Research) diluted 1:300, or monkey anti-S. flexneri convalescent-phase antiserum (Edwin Oaks, Walter Reed Army Institute of Research) diluted 1:1,000. Blots were developed with a Pierce enhanced chemiluminescence (ECL) detection kit (Thermo Fisher).

### RNA isolation.

S. flexneri was grown in the indicated growth medium to late log phase. Four milliliters of late-log-phase cell suspension was mixed with 1 mL of an ice-cold solution containing 95% ethanol and 5% phenol (pH 4.5) and then kept on ice until ready for further processing. Once all samples were ready, the cells were pelleted by centrifugation, resuspended in 100 μL of 1-mg/mL lysozyme in Tris-EDTA (TE) buffer, and incubated at room temperature for 5 min. One milliliter of RNA-Bee (Tel-Test Inc.) was added to the lysozyme-treated cells, 200 μL of chloroform was added, and then the solution was centrifuged at 4°C and 21,400 × *g* for 15 min. The aqueous phase was collected, mixed with an equal volume of isopropanol, and stored at −80°C overnight. Samples were centrifuged at 21,400 × *g* for 20 min to pellet precipitated RNA, which was then washed once with ice-cold 75% ethanol, air dried, resuspended in water, and DNase treated per the manufacturer’s instructions (Turbo DNA-*free*; Invitrogen).

### cDNA synthesis and qPCR.

Two micrograms of RNA was reverse transcribed into cDNA using the Superscript III kit (Thermo Fisher) per the manufacturer’s instructions. Primers for quantitative PCR (qPCR) were designed using Primer3 (http://bioinfo.ut.ee/primer3-0.4.0/). For real-time qPCR, cDNA was diluted 1:10 and used with Power SYBR green (Thermo Fisher). The qPCR was run on an Applied Biosystems ViiA7 instrument as previously described (81). Relative expression of virulence genes was calculated by using the threshold cycle (ΔΔ*C_T_*) method and normalized to the mean for two reference genes, *secA* and *gyrA*.

### Fractionation of B. thetaiotaomicron CM.

B. thetaiotaomicron CM was fractionated by size using 10-kDa- and 100-kDa-molecular-weight-cutoff centrifugal filters (Amicon). Filters were rinsed once with sterile saline. Then, 2.5 mL of B. thetaiotaomicron CM or BHIS was added to the filter and centrifuged at 3,000 × *g* at 4°C until all but 100 μL of liquid had flowed through the filter. The flowthrough was mixed 1:1 with BHIS, while the top fraction was resuspended in 5 mL of BHIS for S. flexneri growth. For ultracentrifugation, 2.5 mL of BHIS and B. thetaiotaomicron CM was centrifuged at 135,000 × *g* for 2 h. The supernatant was collected. The resulting pellet was resuspended in 1 mL of sterile saline and centrifuged a second time. The supernatant was mixed 1:1 with fresh BHIS, while the pellet was resuspended in 5 mL of fresh BHIS for S. flexneri growth.

### Proteinase K treatment of B. thetaiotaomicron CM.

BHIS and B. thetaiotaomicron CM were treated with 50 μg/mL of proteinase K at 37°C for 3.5 h. Proteinase K-treated BHIS and B. thetaiotaomicron CM were then each mixed 1:1 with fresh BHIS for S. flexneri growth.

### Isolation and normalization of total lipids.

Stationary-phase B. thetaiotaomicron was pelleted by centrifugation for 10 min at 13,000 × *g*. Alternatively, large-scale preparations of crude B. thetaiotaomicron OMVs were isolated by centrifuging 75 mL of B. thetaiotaomicron CM for 3.5 h at 38,400 × *g*. Total B. thetaiotaomicron lipids from either cell pellets or crude OMVs were extracted by the method of Bligh and Dyer (82). For a negative control, the crude OMV isolation and lipid extraction were performed starting with 75 mL of uninoculated BHIS to generate a sample referred to as “mock lipids.” E. coli total lipid extracts were purchased (Avanti Polar Lipids). Extracted or purchased lipids were dried under a stream of nitrogen and then stored at −20°C until use.

For relative quantitation, lipids were resuspended in saline and then normalized by their fluorescence in the presence of the lipophilic fluorescent dye FM4-64 FX by a method adapted from a previously published protocol (83). Briefly, 2-fold serial dilutions of the lipid samples were made, and 50 μL of each dilution was mixed with 150 μL of 6.67 μg/mL FM4-64 FX (final concentration, 5 μg/mL) and incubated for 10 min at room temperature. Fluorescence was measured (excitation [Ex]/emission [Em], 510/640 nm) on a FlexStation 3 plate reader. Serial dilutions falling within the linear range of the assay were used to determine relative concentrations of the lipid preparations. To standardize lipid concentrations, lipid samples were adjusted with saline, such that a 50-μL aliquot gave a signal of 240 relative fluorescent units (RFU) in a 200-μL reaction mixture (5 μg/mL FM4-64 FX). To assess the effect of lipids on S. flexneri IpaC levels, normalized lipid stocks (240 RFU) were diluted 6-fold in S. flexneri growth medium for a final lipid concentration of 40 RFU.

### OMV isolation.

To isolate outer membrane vesicles (OMVs), 240 mL of B. thetaiotaomicron CM, prepared as described above, was concentrated to 7 mL using either 100-kDa-cutoff centrifugal filters (Amicon) or a tangential flow filtration device with a 100-kDa-cutoff filter (Vivaflow 50R; Sartorius). Concentrated B. thetaiotaomicron CM was then ultracentrifuged at 135,000 × *g* for 2 h to pellet crude B. thetaiotaomicron OMVs. Depending on the downstream application, the crude OMVs were either washed once with saline or further purified on an OptiPrep (Sigma) density gradient modified from a published protocol (84). Briefly, the crude OMVs were resuspended in 45% (vol/vol) OptiPrep and added to the bottom of an Ultraclear (Beckman) centrifuge tube. Five layers of OptiPrep, decreasing in increments of 5% each layer down to 20% OptiPrep, were layered on top of the OMVs. The OMVs were then ultracentrifuged at 4°C for 20 h at 150,000 × *g*. The density gradient was collected in 12 fractions, and SDS-PAGE was performed to identify the fractions that contained OMVs. OMV-containing fractions were then pooled and diluted in 10-fold-excess Dulbecco’s phosphate-buffered saline supplemented with salts (DPBS) (0.2 g/L KCl, 0.2 g/L KH_2_PO_4_, 11.7 g/L NaCl, 1.15 g/L Na_2_PO_4_, 0.1 g/L MgCl_2_·6H_2_O, and 0.1 g/L CaCl_2_), and the OMVs were collected by centrifugation at 38,400 × *g* for 3.5 h. The entire OMV isolation protocol was also performed on 240 mL of sterile BHIS to generate a sample for negative controls, referred to as mock extract.

### IM/OM preparations.

To isolate inner and outer membranes (IM and OM, respectively), a stationary-phase culture of the indicated B. thetaiotaomicron strain was centrifuged at 13,000 × *g* for 10 min. The resulting pellet was resuspended in buffer (10 mM Na_2_HPO_4_ and 5 mM MgSO_4_), lysed by sonication, and then centrifuged at 13,000 × *g* to remove cell debris. Total membrane (TM) was isolated by centrifuging the supernatant at 135,000 × *g* for 40 min. To separate inner and outer membranes, the total membrane pellet was resuspended in 1% (wt/vol) *N*-lauroyl sarcosine (Sarkosyl) and incubated with rocking at room temperature for 1 h. To separate IM from OM, the sample was centrifuged at 135,000 × *g* for 40 min. The Sarkosyl-soluble supernatant, which contained the inner membrane fraction, was collected. The Sarkosyl-insoluble pellet, which contained the outer membrane, was washed with 1% Sarkosyl, centrifuged at 135,000 × *g* for 40 min, and then resuspended in water for downstream applications.

### Quantification of membrane proteins.

For quantification of protein, total membranes, outer membranes, or OMVs were resuspended in 0.5% Triton X-100, while inner membranes were resuspended in 1% *N*-lauroyl sarcosine. Protein concentration was quantified by the DC protein assay (Bio-Rad) per the manufacturer’s instructions. For all experiments involving OMVs, OMV concentrations are normalized by the total protein concentration of the vesicles.

### TEM.

Five microliters of OMVs that had been diluted 1:10 in saline were added to a glow-discharged 200-mesh Formvar/carbon grid (Electron Microscopy Sciences; FCF200-Cu) and incubated for 5 min. Excess sample was wicked off, and the grid was washed once with a drop of water and stained for 1 min with 2% uranyl acetate before being air dried and imaged. Imaging was performed on an FEI Tecnai Spirit transmission electron microscope (TEM) at the Center for Biomedical Research Support Microscopy and Imaging Facility at UT Austin (RRID no. SCR_021756).

### Expression of His-tagged proteins in B. thetaiotaomicron.

*Bacteroides* expression vector pFD340 (85) expressing His-tagged membrane proteins was transformed into the donor strain E. coli S17-1 *λpir* and conjugated into B. thetaiotaomicron as previously described (86). Briefly, pellets from overnight cultures of E. coli S17-1 *λpir* expressing the tagged gene of interest and B. thetaiotaomicron were pooled, plated on BHI agar plates, and grown aerobically overnight at 37°C without selection. The resulting lawn was resuspended in a small volume of BHIS. To recover single colonies of B. thetaiotaomicron conjugants, dilutions were plated on BHI agar containing gentamicin and erythromycin and incubated anaerobically at 37°C for 24 h.

### OMV uptake assay.

To assess fusion of B. thetaiotaomicron OMVs by S. flexneri, a slight modification of a previously published protocol was used (87). Briefly, 50 μg of purified B. thetaiotaomicron OMVs in 250 μL of saline was stained with 5 μg/mL of FM4-64 FX at room temperature for 15 min. To eliminate excess unbound dye, the stained OMVs were washed three times with a 20-fold excess volume of saline on a centrifugal filter (100-kDa-molecular-weight cutoff; Amicon). To control for dye carryover, the staining and washing procedure described above was also performed on 250 μL of saline in the absence of OMVs. To measure OMV uptake, S. flexneri from a late-log-phase culture was adjusted to an OD_650_ of 1.0 in 1 mL of saline and coincubated, with rocking at room temperature, with either 50 μg of stained OMVs or an equal volume of stained saline in the absence of OMVs. At the indicated time points, 500 μL of sample was removed, washed three times with saline, fixed for 10 min in 4% paraformaldehyde, and washed three times more. The fluorescence (Ex/Em of 510/640 nm; SpectraMax M3) and OD_650_ (FlexStation 3) of S. flexneri that had been coincubated with stained OMVs were read on the respective microplate readers. To control for dye diffusion from OMVs, the OMVs were stained as described above and then soaked in 1 mL of saline for 30 min. After 30 min the soaked OMVs were separated by centrifugation at 135,000 × *g* for 1 h. The supernatant and soaked OMVs were each collected and used in an OMV uptake assay as described above.
