# Supplementary material for: Bacteroides thetaiotaomicron Outer Membrane Vesicles Modulate Virulence of Shigella flexneri
Source: mBio. 2022 Sep 14;13(5):e02360-22. doi: 10.1128/mbio.02360-22 (PMC9600379; doi:10.1128/mbio.02360-22)
Supplement: FIG S1 [file mbio.02360-22-s0001.docx]

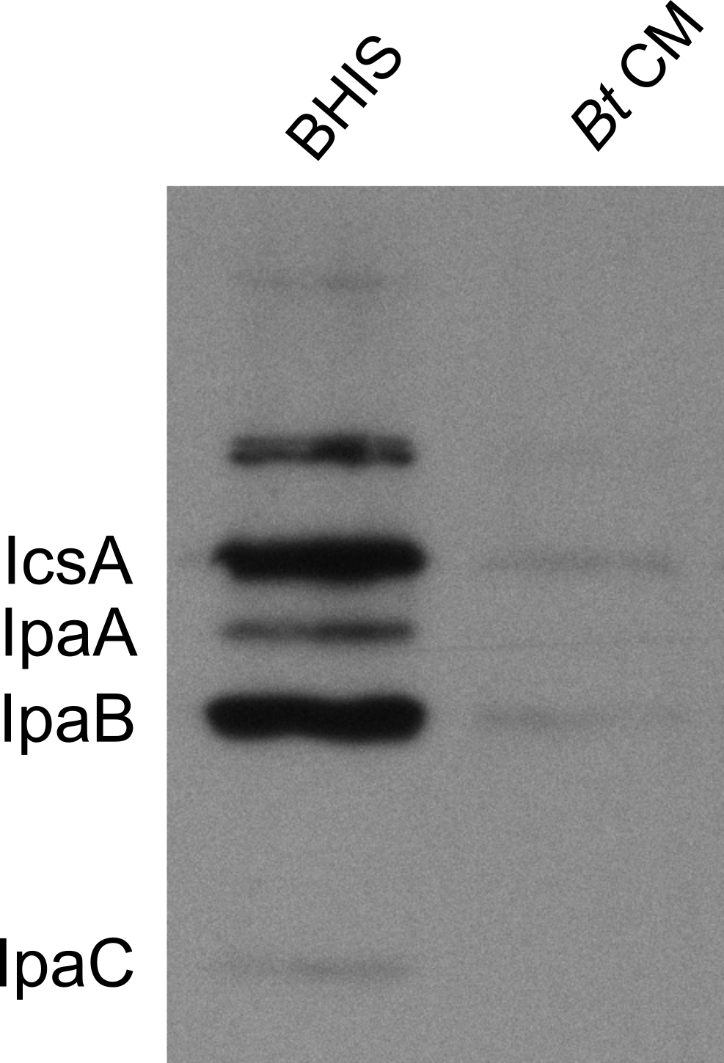


Figure S1: *Bt* CM reduces *S. flexneri* virulence factor secretion. Secreted proteins were collected from *S. flexneri* grown in either BHIS or ½ *Bt* CM. A Western blot using monkey anti- *Shigella* convalescent-phase antiserum was performed to look at the relative secretion of a panel of virulence factors. A representative Western blot of 5 independent replicates is shown.
