# Supplementary material for: Bacteroides thetaiotaomicron Outer Membrane Vesicles Modulate Virulence of Shigella flexneri
Source: mBio. 2022 Sep 14;13(5):e02360-22. doi: 10.1128/mbio.02360-22 (PMC9600379; doi:10.1128/mbio.02360-22)
Supplement: FIG S2 [file mbio.02360-22-s0002.docx]

Figure S2: *E.coli* CM does not affect *S. flexneri* virulence gene expression*.* Relative virulence gene expression of *S. flexneri* grown to log phase in either BHIS or ½ *E.coli* CM was measured by RT-qPCR. The *E. coli* CM was tested in parallel with the BT CM shown in Fig. 3B, and the BHIS control values are the same as shown in that figure. *C_t_* values were normalized to the mean of two endogenous controls, *gyrA* and *secA*. *P* values were determined from the Δ*C_t_* values of three biological replicates using a two-tailed Student’s *t* test with Holm-Šídák correction for multiple comparisons. Error bars indicate SD.
