# Supplementary material for: Bacteroides thetaiotaomicron Outer Membrane Vesicles Modulate Virulence of Shigella flexneri
Source: mBio. 2022 Sep 14;13(5):e02360-22. doi: 10.1128/mbio.02360-22 (PMC9600379; doi:10.1128/mbio.02360-22)
Supplement: FIG S3 [file mbio.02360-22-s0003.docx]

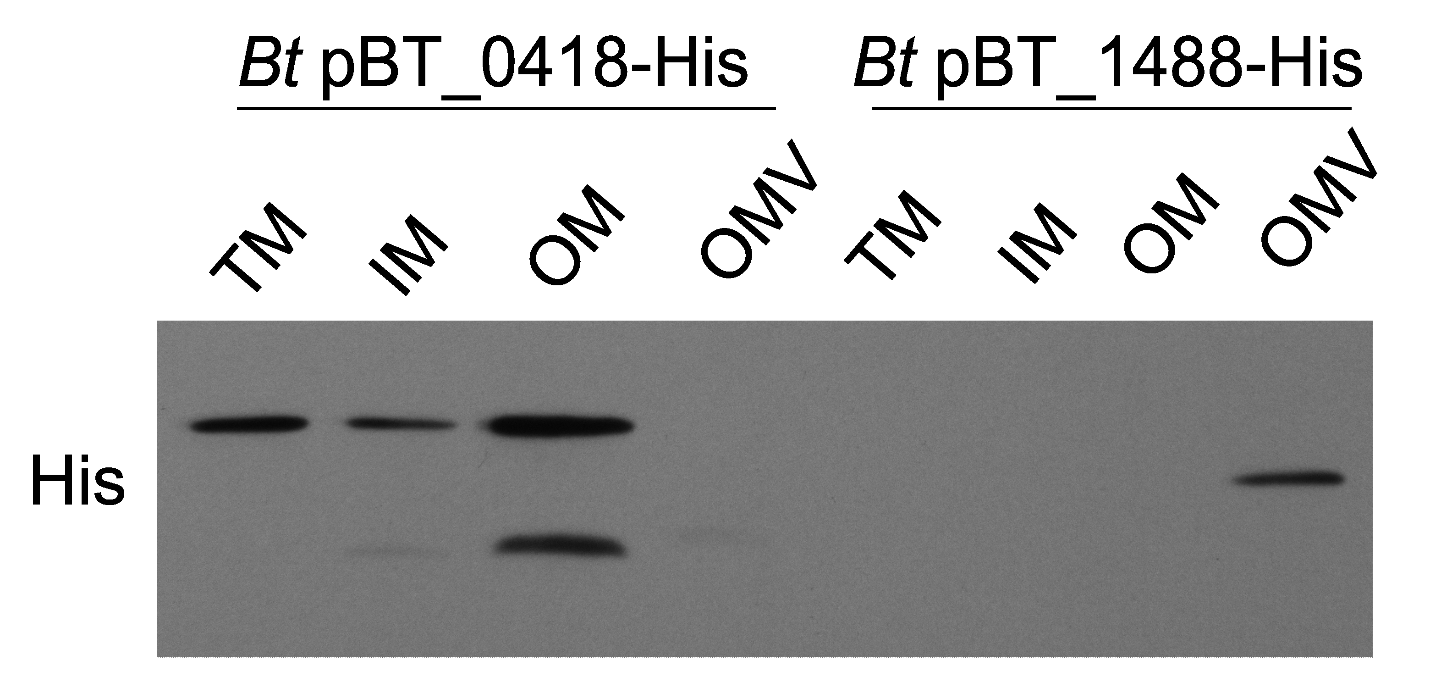


Figure S3: *Bt* OMVs have the expected protein profile: BT_0418 is localized to the outer membrane, while BT_1488 is localized to outer membrane vesicles. Total membrane (TM), inner membrane (IM), outer membrane (OM) or outer membrane vesicles (OMV) were extracted from *Bt::*pFD340/BT_0418-6xHis and *Bt*::pFD340/BT_1488-6xHis strains. Membrane preps were normalized using a DC protein assay, and 10 µg of sample was loaded per well for an anti-His Western blot.
