# Supplementary material for: Bacteroides thetaiotaomicron Outer Membrane Vesicles Modulate Virulence of Shigella flexneri
Source: mBio. 2022 Sep 14;13(5):e02360-22. doi: 10.1128/mbio.02360-22 (PMC9600379; doi:10.1128/mbio.02360-22)
Supplement: FIG S4 [file mbio.02360-22-s0004.docx]

Figure S4: Direct contact between *Bt* OMVs and *S. flexneri* is required for dye transfer. *Bt* OMVs were stained with 5 µg/mL of the fluorescent dye FM4-64 FX, washed extensively, soaked in saline for 30 minutes, and the separated out of their soaking solution. *S. flexneri* was incubated in the OMV soaking solution, the presence of soaked OMVs, or the presence of unsoaked stained OMVs. At a timepoint 2 hours, *S. flexneri* fluorescence was measured on a plate reader (Ex/Em of 510/640 nm). *P* values were determined from three biological replicates using a one-way Anova with Dunnett’s test for multiple comparisons (*, *p* < 0.05). Error bars indicate standard error.
