# Supplementary material for: Bacteroides thetaiotaomicron Outer Membrane Vesicles Modulate Virulence of Shigella flexneri
Source: mBio. 2022 Sep 14;13(5):e02360-22. doi: 10.1128/mbio.02360-22 (PMC9600379; doi:10.1128/mbio.02360-22)
Supplement: TABLE S1 [file mbio.02360-22-s0005.docx]

**Table S1.** Strains and plasmids used in this study.

| **Strain or plasmid** | **Description** | **Reference/Source** |
| --- | --- | --- |
| ***E. coli*** |  |  |
| DH5α | Cloning strain | (1) |
| MG1655 | Wild type | (2) |
| *E. coli* s17-1 λpir | Conjugation donor strain | (3) |
| ***S. flexneri* 2a** |  |  |
| 2457T | Wild type, serotype 2a | Walter Reed Army Institute of Research (4) |
| ***Bacteroides theatiotaomicron*** |  |  |
| VPI 5482 | Wild type, Gent^R^, Erm^S^ | ATCC 29148 |
| **Human** |  |  |
| Henle | HeLa contaminant cell line | ATCC CCL-6 (intestine 407) |
| **Plasmids** |  |  |
| pWKS30 | Low-copy expression vector | (5) |
| p*virF* S-tag | *virF*_S-tag in pWKS30 | This study |
| pFD340 | *Bacteroides* expression vector | (6) |
| pFD340/0418-6xHis | BT_0418-6xHis in pFD340 | (7) |
| pFD340/1488-6xHis | BT_1488-6xHis in pFD340 | (7) |

1. Hanahan D. 1983. Studies on transformation of *Escherichia coli* with plasmids. J Mol Biol 166:557–580.

2. Blattner FR, Plunkett G, Bloch CA, Perna NT, Burland V, Riley M, Collado-Vides J, Glasner JD, Rode CK, Mayhew GF, Gregor J, Davis NW, Kirkpatrick HA, Goeden MA, Rose DJ, Mau B, Shao Y. 1997. The complete genome sequence of *Escherichia coli* K-12. Science 277:1453–1462.

3. de Lorenzo V, Timmis KN. 1994. Analysis and construction of stable phenotypes in gram-negative bacteria with Tn5- and Tn10-derived minitransposons. Methods Enzymol 235:386–405.

4. Wei J, Goldberg MB, Burland V, Venkatesan MM, Deng W, Fournier G, Mayhew GF, Plunkett G, Rose DJ, Darling A, Mau B, Perna NT, Payne SM, Runyen-Janecky LJ, Zhou S, Schwartz DC, Blattner FR. 2003. Complete genome sequence and comparative genomics of *Shigella flexneri* serotype 2a strain 2457T. Infect Immun 71:2775–2786.

5. Wang RF, Kushner SR. 1991. Construction of versatile low-copy-number vectors for cloning, sequencing and gene expression in *Escherichia coli*. Gene 100:195–199.

6. Smith CJ, Rogers MB, McKee ML. 1992. Heterologous gene expression in *Bacteroides fragilis*. Plasmid 27:141–154.

7. Valguarnera E, Scott NE, Azimzadeh P, Feldman MF. 2018. Surface exposure and packing of lipoproteins into outer membrane vesicles are coupled pProcesses in *Bacteroides*. mSphere 3:e00559-18.
