# Supplementary material for: Bacteroides thetaiotaomicron Outer Membrane Vesicles Modulate Virulence of Shigella flexneri
Source: mBio. 2022 Sep 14;13(5):e02360-22. doi: 10.1128/mbio.02360-22 (PMC9600379; doi:10.1128/mbio.02360-22)
Supplement: TABLE S2 [file mbio.02360-22-s0006.docx]

Table S2. Primers used in this study.

| Purpose | Primer | Sequence (5’ to 3’) |
| --- | --- | --- |
| RT-qPCR | RT-virF_F | tctgaggagaaaagggggtta |
|  | RT-virF_R | acgccatctcttctcgatgt |
|  | RT-virB_F | tgccagaaaactagcaaacg |
|  | RT-virB_R | gttctgacgcgattggaaat |
|  | RT-ipaB_F | ggcagctgttggtttagctc |
|  | RT-ipaB_R | cgactgctgcaactaggaca |
|  | RT-ipaC_F | cattgctcgccttactgaca |
|  | RT-ipaC_R | atcgaacgcaatcaatgaca |
|  | RT-icsA_F | tgatggtaatagttgtggcgg |
|  | RT-icsA_R | gattgtctcctgtaacagcctc |
|  | RT-gyrA_F | atgaactgatggccgatctc |
|  | RT-gyrA_R | atgtgttccatcagcccttc |
|  | RT-secA_F | gcgtaccggtgaaggtaaaa |
|  | RT-secA_R | tcaggccaaggaattcaaac |
| Amplify and S-Tag VirF | Upstream virF-Stag_F | ctagtgaattcgatttgtggagcctccag |
|  | Upstream virF-Stag_R | cagcggtttctttaccagactcgagaaattttttatgatataagtaaaatttctttggag |
|  | Downstream virF-Stag_F | gtctggtaaagaaaccgctgctgcgaaatttgaacgccagcacatggactcgtctactagcgcagcttaaatgcttcatagcccatc |
|  | Downstream virF-Stag_R | actgtgtcgacaaaaaaaaacccatctggcaatag |
